# An oncogenic splice variant of PDGFRα in adult glioblastoma as a therapeutic target for selective CDK4/6 inhibitors

**DOI:** 10.1038/s41598-022-05391-9

**Published:** 2022-01-24

**Authors:** Taiji Hamada, Toshiaki Akahane, Seiya Yokoyama, Nayuta Higa, Mari Kirishima, Kei Matsuo, Michiko Shimokawa, Koji Yoshimoto, Akihide Tanimoto

**Affiliations:** 1 Department of Pathology, Kagoshima University Graduate School of Medical and Dental Sciences, 8-35-1 Sakuragaoka, Kagoshima, 890-8544 Japan; 2 Department of Neurosurgery, Kagoshima University Graduate School of Medical and Dental Sciences, Kagoshima, Japan; 3 Center for Human Genome and Gene Analysis, Kagoshima University Hospital, Kagoshima, Japan

**Keywords:** Cancer, Genetics, Molecular medicine, Oncology

## Abstract

Understanding human genome alterations is necessary to optimize genome-based cancer therapeutics. However, some newly discovered mutations remain as variants of unknown significance (VUS). Here, the mutation c.1403A > G in exon 10 of the platelet-derived growth factor receptor-alpha (*PDGFRA*) gene, a VUS found in adult glioblastoma multiforme (GBM), was introduced in human embryonal kidney 293 T (HEK293T) cells using genome editing to investigate its potential oncogenic functions. Genome editing was performed using CRISPR/Cas9; the proliferation, drug sensitivity, and carcinogenic potential of genome-edited cells were investigated. We also investigated the mechanism underlying the observed phenotypes. Three GBM patients carrying the c.1403A > G mutation were studied to validate the in vitro results. The c.1403A > G mutation led to a splice variant (p.K455_N468delinsN) because of the generation of a 3’-acceptor splice site in exon 10. *PDGFRA*-mutated HEK293T cells exhibited a higher proliferative activity via PDGFRα and the cyclin-dependent kinase (CDK)4/CDK6-cyclin D1 signaling pathway in a ligand-independent manner. They showed higher sensitivity to multi-kinase, receptor tyrosine kinase, and CDK4/CDK6 inhibitors. Of the three GBM patients studied, two harbored the p.K455_N468delinsN splice variant. The splicing mutation c.1403A > G in *PDGFRA* is oncogenic in nature. Kinase inhibitors targeting PDGFRα and CDK4/CDK6 signaling should be evaluated for treating GBM patients harboring this mutation.

## Introduction

The development of new technologies, such as next-generation sequencing (NGS), has led to the accumulation of annotation data related to cancer genomics^1–4^. In fact, these databases are being continually updated with data with translational implications, thus allowing improved cancer diagnosis, prognosis prediction, and treatment with molecularly targeted drugs. The value of comprehensive genome analyses was recognized for brain tumors after the introduction of a new integrative molecular diagnosis strategy for tumor classification by the World Health Organization^5^. However, the pathogenic or oncogenic significance remains unknown for several newly found clinical variants, known as variants of unknown significance (VUS).

In our previous study, we reported a rare variant of the gene encoding the platelet-derived growth factor receptor-alpha (*PDGFRA*) using NGS in IDH1 wild-type glioblastoma multiforme (GBM)^6^. This VUS is a member of a possible Japanese (or Asian) genotype group manifested by *PDGFRA* gene amplification and mutation, but without *TERT* promoter mutations. As the availability of molecularly targeted drugs for brain tumors is limited, the elucidation of oncogenic functions of VUS, including in the *PDGFRA* gene, is essential for the development of new drugs.

The standard methods to evaluate the oncogenic potential of newly discovered variants include computational prediction and in vivo and in vitro studies^7–11^. Some in vivo studies have been performed on genetically modified mice harboring mutations in target genes based on the information obtained from the clinical sequences or on results obtained in vitro^12^. Therefore, in vitro experiments are essential to generate primary information regarding the functions of VUS. Usually, normal or cancer cells (both primary cells and cell lines) of human or non-human origin are used for in vitro studies. The transfer of specific genes into cultured cells can be achieved via plasmids, viral vectors, or genome editing approaches^13, 14^. Despite the limitation of the availability of ideal cells, genome editing remains the most ideal method to introduce gene mutations in cells^15^. However, plasmid-mediated gene transfer remains a useful method and is often used to clarify the function of gene mutations in cancer^16^.

In the present study, we introduced a c.1403A > G variant in exon 10 of the *PDGFRA* gene in human embryonal kidney 293 T (HEK293T) cells via genome editing to elucidate its role. We evaluated the morphology and proliferation of gene-edited cells as well as their sensitivity to molecularly targeted drugs, such as CDK4/6 inhibitors. Moreover, we analyzed three patients with GBM carrying the c.1403A > G mutation. This study might provide valuable insights regarding the potential of genome editing-based in vitro studies for the functional evaluation of VUS.

## Results

### Genome sequencing and pathological analysis of GBM cases

**Figure 1:**
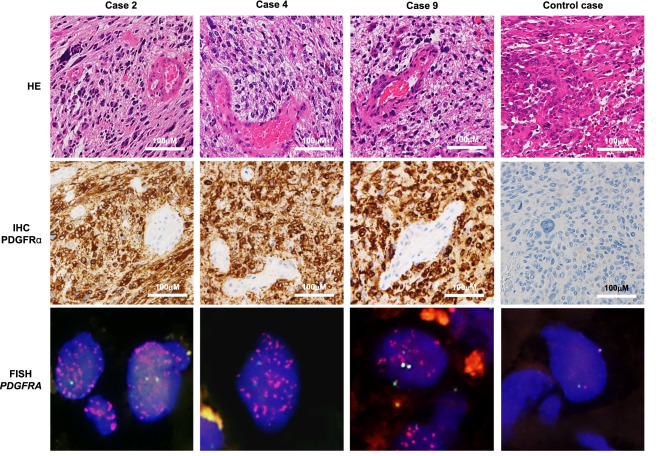
Representative histology, immunohistochemistry (IHC), and fluorescence in situ hybridization (FISH) of GBM cases with the *PDGFRA* c.1403A > G mutation. Representative histology, PDGFRα immunohistochemistry, and *PDGFRA* FISH images of GBM cases are shown. The H&E sections demonstrate a typical GBM morphology consisting of a dense proliferation of highly atypical tumor cells with high vascularity and palisading necrosis. PDGFRα was diffusely positive in the cytoplasm and FISH showed the amplification of the PDGFRA gene (green, centromere; red, *PDGFRA* gene) in *PDGFRA*-mutated cells but not in wild-type cells. The control case is a GBM without PDGFRA amplification and mutations.

We recently reported the existence of a subgroup of patients harboring mutations in the *PDGFRA* gene (without *TERT* promoter mutations)^6^; 11 out of 64 GBM cases (17%) harbored wild-type IDH1 and showed no 1p19q co-deletion (Table S1). Two cases (cases nos. 3 and 10) with mutations in the intracytoplasmic kinase domain showed no *PDGFRA* amplification, while the remaining nine cases harbored mutations in the extra-cytoplasmic domain and exhibited 30- to 84-fold gene amplification; most of the *PDGFRA* mutations were VUS or not registered in the JAX and OncoKB databases. Of note, three cases (case nos. 2, 4, and 9) harbored a c.1403A > G mutation in both alleles of exon 10 of *PDGFRA*. This variant is rarely reported in adult GBM cases, and the COSMIC database annotates the c.1403A > G mutation as a missense mutation (p. N468S). The representative GBM morphology, PDGFRα IHC, and *PDGFRA* FISH results for these three cases are shown in Fig. 1. H&E staining demonstrated the proliferation of highly atypical and pleomorphic tumor cells with vascular proliferation and palisading necrosis. Additionally, immunoreactive PDGFRα was diffusely located in the cytoplasm of *PDGFRA*-mutated cases, but not in wild-type cases. FISH studies showed a marked amplification of the *PDGFRA* gene in Cases 2, 4, and 9 with *PDGFRA* mutation. Therefore, we decided to investigate the *PDGFRA* c.1403A > G mutation via genome editing in vitro.

### Selection of sgRNAs

To introduce double-strand breaks (DSB) near the mutated *PDGFRA*, six sgRNAs were designed using CRISPRdirect^17^ (Fig. S1). The cleavage efficiency of each sgRNA was evaluated using the SSA^18^ and T7E1 assays. The T7E1 assay showed comparable cleavage efficiency for all sgRNAs, except for sgRNA6 (Fig. S2A). Additionally, the SSA assay revealed that sgRNA3 and sgRNA4 had higher activities (Fig. S2B). Since the distance between the editing site and DSB should be less than 30 bp in HR-based genome editing^19^, the sgRNA4, which is 15 bp apart from the editing site (versus 44 bp for sgRNA3), was used thereafter.

### Establishment of *PDGFRA*-mutated HEK293T cells using the microhomology-assisted excision (MhAX) method

**Figure 2:**
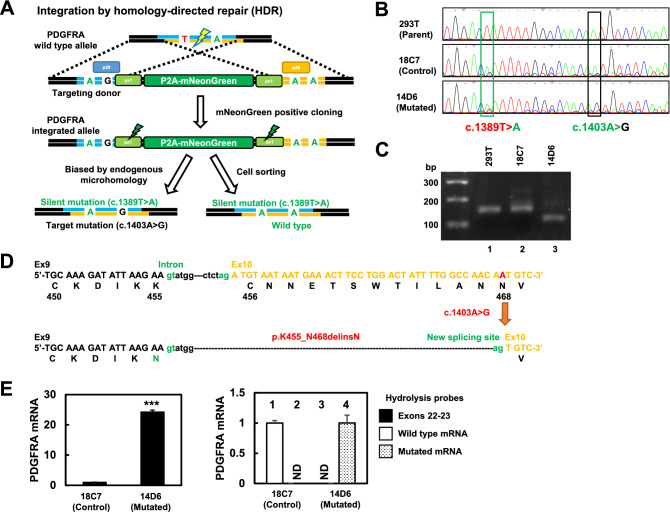
Schematic presentation of the genome editing strategy used in this study and effects of the *PDGFRA* c.1403A > G mutation. **(A)** A scheme representing the approach used for the generation of c.1403A > G *PDGFRA*-mutated HEK293T cells. Arrows indicate the primers designed to amplify the predicted insertion site with (product size: 1091 bp) and without (product size: 149 bp) the selection cassette. The figure is drawn using Microsoft PowerPoint 2019 MSO (https://support.office.com/ja-jp/article/Office). (**B)** Sanger sequencing of the edited clones after selection. Sequencing of the 14D6 clone showed both the targeted (c.1403A > G) and silent (c.1389 T > A) mutations. Sequencing of the 18C7 clone showed only the silent mutation. (**C)** Agarose gel analysis of *PDGFRA* mRNA expression using RT-PCR in the established clones. The size of the PCR products from the 14D6 clone was smaller than those from parental HEK293T and 18C7 isogenic control cells. (**D)** Schematic presentation of the c.1406A > G *PDGFRA*-associated new splicing site leading to the deletion of 39 nucleotides (13 amino acids). (**E)** *PDGFRA* mRNA expression analysis by qRT-PCR (n = 6 per group). Left panel: in qRT-PCR recognizing exons 22 to 23, the 14D6 clone exhibited an increase in mRNA expression compared to that in the 18C7 isogenic control clone. Right panel: using probes for the detection of the splicing mutation (lanes 2 and 4) and wild-type (lanes 1 and 3) sequences, mutated cDNA was amplified only in the 14D6 clone (lane 4), whereas wild-type cDNA was only detected in the 18C7 clone (lane 1). *GAPDH* was used as the reference gene for quantification and the data were shown in fold changes of expression. ***, p < 0.001; ND, Not detected. For uncropped gel source of **(C)**, see Supplemental Fig. S11.

The MhAX method^20^ was used to generate HEK293T cells harboring the *PDGFRA* c. 1403A > G mutation. Figure 2A summarizes the genome editing strategy used. After co-transfection of the sgRNA4- and Cas9 nuclease-expressing vector and of the donor vector into HEK293T cells, mNeonGreen-positive cells were retrieved by cell sorting; 44 clones were obtained after sorting 72 single cells (Fig. S3). Gene targeting was confirmed by PCR genotyping; 1.1 kbp bands were amplified in the clones containing the selection cassette (Fig. S4). Sanger sequencing revealed that the selection cassette was correctly inserted in the 1D3 clone; therefore, we used it for the second editing round (Fig. S5). The ps1 site-targeted Cas9 vector was introduced into 1D3 cells and the selection cassette was deleted out of the *PDGFRA* gene. Next, mNeonGreen-negative cells were sorted and cells harboring the target gene mutation as well as isogenic controls were obtained from 131 single-cell clones (Fig. S6). After PCR-based genotyping (Fig. S7) and Sanger sequencing (Fig. 2B), clone 14D6 (harboring c.1403A > G and c.1389 T > A) and clone 18C7 (the isogenic control, harboring only c.1389 T > A) were selected, expanded, and subjected to deep sequencing by NGS; analysis was performed using CRISPResso2^21^. Importantly, both the clones showed the expected mutations with nearly 85% of the sequence read ratio.

### *PDGFRA* mRNA expression in the established HEK293T cell clones

Next, we examined the mRNA expression of *PDGFRA* in the clones using paired primers designed to include the mutation site (c.1403A > G). The PCR product from the 14D6 clone (lane 3) was shorter than that from the 18C7 clone (lane 2) and parental HEK293T cells (lane 1) (Fig. 2C). Of note, when these PCR products were analyzed by Sanger sequencing, the first 39 bp of exon 10 were deleted in clone 14D6 (Fig. S8). We speculated that the c.1403A > G mutation generated a new 3’-splice site, resulting in intron elongation, in-frame splicing, and consequently, a new *PDGFRA* splice variant (p.K455_N468delinsN) (Fig. 2D). Additionally, to measure the *PDGFRA* mRNA levels, quantitative RT-PCR was performed using primers and a hydrolyzed probe recognizing exons 22 to 23. The 14D6 mutated clone exhibited increased *PDGFRA* mRNA expression compared to that in the 18C7 isogenic control clone (Fig. 2E, left panel). Notably, using hydrolyzed probes for the detection of either the splicing mutation (columns 2 and 4) or wild-type cDNA (columns 1 and 3), only mutated cDNA was amplified from the 14D6 clone (column 4), whereas only wild-type cDNA was detected in the 18C7 clone (column 1) (Fig. 2E, right panel).

We tried to establish human GBM cells harboring *PDGFRA* c. 1403A > G mutation using U-251MG and KNS-42 cell lines. However, the KNS-42 cells with and without the *PDGFRA* c. 1403A > G mutation did not show clonal expansion after genome editing procedure. The U-251MG cells exhibited clonal expansion and mRNA expression of the splice variant (p.K455_N468delinsN), but did not sufficiently express PDGFRα protein. Thus, we could not obtain GBM cells expressing p.K455_N468delinsN PDGFRα that were available for further study even after the successful genome editing. At present, we do not know the exact reason why these GBM cells are unavailable for the establishment of genome-edited cell lines. These results are precisely described in the Supplementary Fig. S9.

### *PDGFRA* mRNA expression in human GBM cases

Since the c.1403A > G mutation caused a splice variant of *PDGFRA* instead of a missense mutation in vitro, we decided to further study the three GBM cases harboring the c.1403A > G mutation. Notably, in two out of three cases (Cases 2 and 4 in Table S1), mRNA sequences revealed that the c.1403A > G mutation led to an in-frame *PDGFRA* splice variant in line with our in vitro results (Fig. S10). Genomic Sanger sequencing (left) showed two peaks representing mutant [G] and wild-type [A] nucleotides, indicating *PDGFRA* heterozygosity in tumor cells or contamination with the genome from healthy cells. In contrast, RT-PCR showed a single-sized band and the cDNA sequence (right) detected the transcript coding for the in-frame internal deletion.

### Proliferation of *PDGFRA*-mutated HEK293T cells

**Figure 3:**
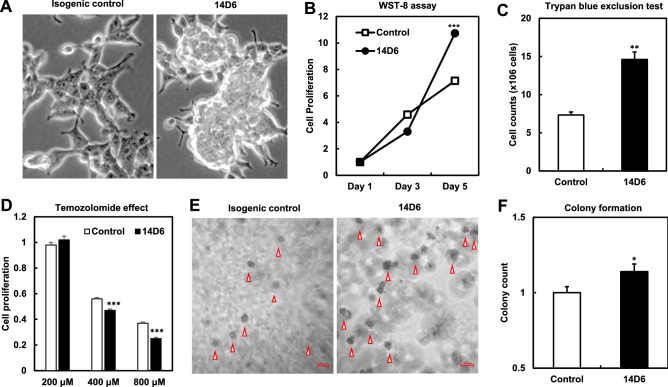
Phenotype analyses of *PDGFRA*-mutated HEK293T cells. **(A)** The14D6 cells showed a round morphology with a high nuclear/cytoplasmic ratio and were arranged in aggregates. (**B,C)** Both the WST assay **(B)** and the trypan blue exclusion test **(C)** disclosed a higher proliferative activity in 14D6 *versus* isogenic control cells. (**D)** The administration of temozolomide caused a higher effect in 14D6 than in isogenic control cells. (**E,F)** The colony-forming assay revealed higher plaque-forming activity in 14D6 cells. Arrowheads indicate the colonies **(E)**. The colony quantification is shown as the count ratio of control to 14D6 cells **(F)**. *, p < 0.05; **, p < 0.01; ***, p < 0.001.

To understand whether the *PDGFRA* splice variant was oncogenic, the proliferation of non-stimulated mutant or isogenic control cells was investigated. Compared with isogenic 18C7 control cells, 14D6 cells showed a round morphology, were arranged in aggregates, and demonstrated a high nuclear/cytoplasmic ratio (Fig. 3A). Moreover, both the WST assay and trypan blue exclusion test after 5 d of culture revealed higher proliferative activity in 14D6 cells (Fig. 3B,C). Additionally, the cell cycle analysis revealed increased cell numbers in the S and G2/M phase and decreased cell numbers in the G0/G1 phase in 14D6 cells compared to 18C7 cells (27% vs. 25%, 23% vs. 18%, and 45% vs. 52%, respectively). Notably, temozolomide (TMZ) was more effective in 14D6 cells (Fig. 3D); additionally, 14D6 cells also exhibited higher plaque-forming activity when compared to isogenic control 18C7 cells (Fig. 3E,F).

### PDGFR signal transduction and activation of the CDK4/CDK6-cyclin D1 pathway in *PDGFRA*-mutated HEK293T cells

**Figure 4:**
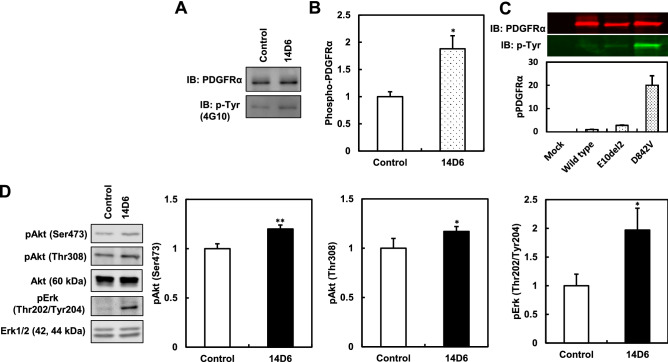
Activation of PDGFR signal transduction pathway-related kinases in *PDGFRA*-mutated HEK293T cells. **(A–C)** The membrane fractions of HEK293T cells were immunoprecipitated using an anti-PDGFRα antibody and subjected to western blotting for the quantification of tyrosine-phosphorylated PDGFRα. After genome editing, the expression of tyrosine-phosphorylated PDGFRα was upregulated in 14D6 cells **(A,B)**. After transfection with PDGFRα expression plasmids, tyrosine-phosphorylation was moderately increased in p.E10del2 (equal to p.K455Ndel13)-transfected, and less expressive in p.D842V-transfected HEK293T cells **(C)**. (**D)** Phosphorylated Akt (pAkt^Ser473^, pAkt^Thr308^) and Erk (pErkThr^202/Tyr204^), downstream targets of PDGFR signal transduction, were increased. *, p < 0.05; **, p < 0.01. The amounts of phosphorylated proteins were normalized to those of total proteins. For uncropped blot source of **(A,C,D)**, see Supplemental Figs. S11 and S12.

**Figure 5:**
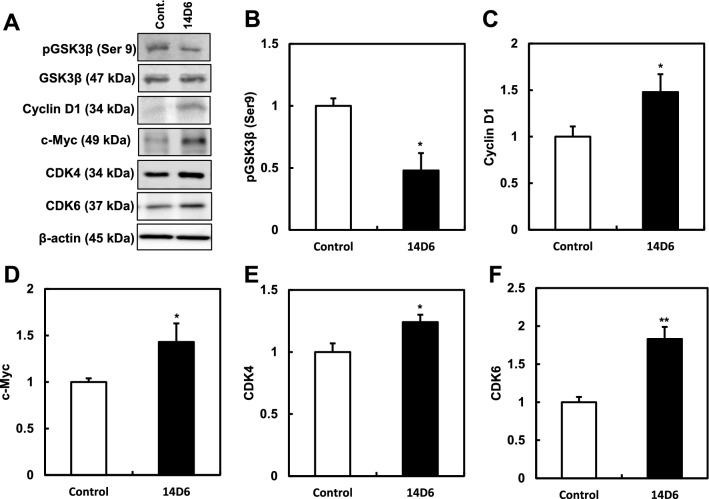
Activation of PDGFR signal transduction pathway-related kinases and targets in *PDGFRA*-mutated HEK293T cells. **(A–F)** The levels of pGSK3β^Ser9^ were decreased in 14D6 cells **(A,B)**. The expression of the PDGFR signal transduction pathway targets, cyclinD1 **(A,C)**, c-Myc **(A,D)**, CDK4 **(A,E)**, and CDK6 **(A,F)**, was increased in 14D6 compared to isogenic control cells. The amounts of phosphorylated proteins were normalized to those of total proteins. *, p < 0.05; **, p < 0.01. For uncropped blot source of **(A)**, see Supplemental Fig. S13.

The expression of PDGFRα in the membrane was monitored by western blotting. The expression of tyrosine-phosphorylated PDGFRα increased in genome-edited 14D6 cells (Fig. 4A,B), indicating an increase in the autophosphorylation of PDGFRα in 14D6 cells. The protein expression of PDGFRα (Fig. 4A) did not increase as much as *PDGFRA* mRNA expression increased (Fig. 2E) in our study condition, but the total PDGFRα protein showed almost the same levels in isogenic control and 14D6 cells. This discrepancy should be clarified further but it is very unknown for us at present. However, the increased expression of phosphorylated-PDGFRα after the genome editing indicates a certain potential of this mutation for tumor proliferation or carcinogenesis. Moreover, the phosphorylation induced by the plasmid-mediated overexpression of PDGFRα was moderately increased but less active in the context of the p.E10del2 (equal to p.K455_N468delinsN)^22^ versus the p.D842V (a constitutively active mutation of the kinase domain) construct (Fig. 4C). Consequently, the downstream components of the PDGFR signal transduction pathway, such as Akt and Erk, showed increased phosphorylation (pAkt^Ser473^, pAkt^Thr308^, and pErkThr^202/Tyr204^), while pGSK3β^Ser9^ was decreased in 14D6 cells (Figs. 4D, 5A,B). Consequently, the expression of cyclinD1, CDK4, and CDK6 (cell cycle regulators of the G1/S phase transition) was also upregulated in 14D6 compared to 18C7 cells, and the expression of c-Myc was also increased (Fig. 5). Collectively, these results suggest that 14D6 cells with the *PDGFRA* c. 1403A > G mutation undergo a faster G1/S transition via the PDGFRα-mediated activation of the CDK4/CDK6-cyclin D1 pathway.

### Impact of molecularly targeted drugs on the proliferation of *PDGFRA*-mutated HEK293T cells

**Figure 6:**
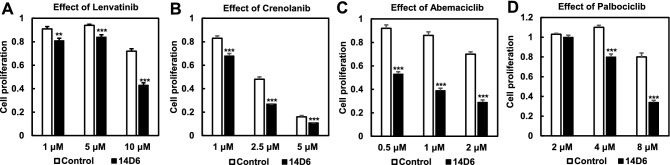
Impact of kinase inhibitors on the proliferation of c.1403A > G *PDGFRA*-mutated HEK293T cells.** (A–D)** The multi-kinase inhibitor, lenvatinb **(A)**, the receptor tyrosine kinase inhibitor crenolanib **(B)**, and the CDK4/CDK6 inhibitors abemaciclib **(C)** and palbociclib **(D)** inhibited the proliferation of 14D6 cells more strongly than that of isogenic control cells.

Remarkably, molecularly targeted drugs, such as the multi-kinase inhibitor lenvatinb, the receptor tyrosine kinase inhibitor crenolanib, and the selective CDK4/CDK6 inhibitors abemaciclib and palbociclib, effectively inhibited the proliferation of 14D6 cells (versus isogenic control cells), in a dose-dependent manner (Fig. 6).

## Discussion

Here, we clearly show that the c.1403A > G mutation produces a novel splicing site, resulting in the in-frame internal deletion and subsequent *PDGFRA* splicing variant p.K455_N468delinsN. This rare splicing variant was previously identified in the context of pediatric high-grade glioma^22^. Furthermore, our in vitro data clearly highlight the oncogenic potential of this variant and its translational implications, since it was detected in two out of three adult cases of GBM harboring the same *PDGFRA* c.1403A > G mutation^6^. Therefore, *PDGFRA* c.1403A < G is probably an oncogenic or pathogenic mutation. Importantly, kinase inhibitors targeting PDGFRα and the CDK4/CDK6 signaling pathway were effective in vitro, and should, therefore, be considered as molecularly targeted therapies for GBM.

The most notable result of this study is the creation of a new 3’-acceptor splice site in exon 10 of *PDGFRA* in the context of the c.1403A < G mutation, and the consequent in-frame internal deletion. We had planned a genome editing-based approach from the beginning. If we had selected a simple plasmid-mediated gene transfer, the expression vector harboring the c.1403A > G substitution would have resulted in the expression of the mutant protein with a single amino acid substitution at position 468 (p.N468S). Therefore, we must stress that the choice of an adequate method is critical in studies aiming to evaluate the functions of gene mutations. Although bioinformatics can be applied to predict splicing mutations, their exact effects should be demonstrated via functional studies^23^. An alternative method would be to use a minigene assay, i.e., the cloning of a target gene with exons and introns into a special plasmid, enabling the analysis of pre-mRNA splicing^24^. Importantly, genome editing allows the reproduction of missense, nonsense, and splicing mutations; however, the availability of competent mammalian cells for editing is limited^15^. Since most of the successful genome editing studies have used HEK293T cells^25^, we selected this cell line to ensure editing efficiency. Since we failed to obtain GBM cell lines harboring c.1403A > G substitution in *PDGFRA*, a use of primary mouse astrocytic cells would be another selection for genome editing study in a future. The use of more relevant neuronal cells, especially primary cultured cells, might show a significant phenotypic change after the introduction of *PDGFRA* mutation as previously reported^22^.

Mutations in the *PDGFRA* and *c-KIT* genes are well documented in gastrointestinal stromal tumors (GIST)^26, 27^. Interestingly, in GIST, *PDGFRA* mutations are mainly located in exon 18, encoding the intracytoplasmic tyrosine kinase domain II. For instance, the most frequent exon 18 p.D842V gain-of-function mutation leads to a constitutive activation of the kinase domain. Additionally, *PDGFRA* mutations were also detected in other cancers, including melanoma, GBM, and colorectal adenocarcinoma^28^. Among them, the *PDGFRA* p.Y288C mutation in GBM leads to the constitutive dimerization and phosphorylation of the extracellular domain in the absence of ligand binding, indicating the constitutive activation of Akt, Erk1/2, and STAT3^22, 29^. However, point mutations in the *PDGFRA* gene leading to new splice variants, such as the one reported in this study, are rare in GBM^30^. As per the classification proposed by Wimmer et al., *PDGFRA* p.K455_N468delinsN is a Type III splicing mutation^31^; these mutations are easily misclassified as synonymous, non-synonymous, and nonsense mutations^23^, according to the databases.

Although the exact mechanism(s) explaining the constitutive activation of the new mutation reported here remains unknown, the deletion of 13 amino acids located at the immunoglobulin-like domain 5, which are necessary for receptor dimerization and activation, may result in the ligand-independent activation of the tyrosine kinase in line with the results observed for other variants^32, 33^. For instance, the deletion of 81 amino acids spanning the immunoglobulin-like domains D4 and D5, via the re-arrangement of exons 8 and 9 (also observed in GBM), is constitutively activated^34^. It is noteworthy that the p.K455_N468delinsN variant is also found in the context of the amplified *PDGFRA* gene, which is observed in 15% of GBM cases^35^; our previous study reported 17% amplification in GBM^6^. However, in pediatric high-grade glioma, *PDGFRA* gene amplification is distinct from that observed in adult GBM^36^. Therefore, the co-occurrence of this splice variant and *PDGFRA* amplification would enhance the downstream signal transduction and subsequent proliferative/oncogenic activity in both adult and pediatric GBM cases.

We clearly showed that multi-kinase (lenvatinib), receptor tyrosine kinase (crenolanib), and CDK4/CDK6 (abemaciclib and palbociclib) inhibitors significantly reduced the proliferation of *PDGFRA-*mutated cells. These molecularly targeted drugs are not specific to PDGFRα; lenvatinib and crenolanib, for example, have wide-ranged pharmacological effects on various types of receptor tyrosine and downstream kinases^29, 37, 38^. However, abemaciclib, and palbociclib specifically target CDK4/CDK6; these and related proteins regulate the G1/S transition and act downstream of the classical signaling pathways induced by growth factors and hormones^39, 40^. In fact, (the amplification or) mutation in the upstream regulators of cyclin D1, CDK4/CDK6, can lead to abnormal cell cycle progression and cell proliferation^41, 42^. Additionally, the activation of CDKs also induces genomic and chromosomal instability, leading to carcinogenesis^43^. Therefore, CDK inhibitors are expected to target dysregulated cell proliferation in various cancers^44^. Importantly, CDK4/CDK6 inhibitors are already used to treat breast cancer^45^ and are being tested in clinical trials as therapeutic agents for other cancers, including GBM showing high CDK4/CDK6 activity^45^. The activated c-Myc signaling pathway is another therapeutic target for reducing the proliferation of *PDGFRA-*mutated cells. However, c-Myc therapeutic targeting remains challenging, even though some approaches via deregulation of transcription, disruption of DNA-binding, and control of post-transcriptional regulation are employed to inactive c-Myc signaling pathway^46^. In contrast, CDK4/6 inhibitor enhances TMZ effect and are expected to overcome TMZ resistance in GBM treatment^47, 48^. Thus, our data support the use of molecularly targeted drugs, including selective CDK4/CDK6 inhibitors for the treatment of GBM^49–51^.

One report showed that GSK3β inhibition results in activation of c-Myc and increase of DNA methylation in O^6^-methylguanine DNA methyltransferase (MGMT) promoter by c-Myc-dependent recruitment of DNA (cytosine-5)-methyltransferase 3A, and, therefore, GSK3β inhibition enhances TMZ effect in GBM cells^52^. This is very consistent with our results that the cells harboring c.1403A > G substitution in *PDGFRA* showed an increased sensitivity to TMZ, c-Myc activation, and GSK3β inactivation. Thus, the GBM cases having the specific *PDGFRA* mutation would be more sensitive to TMZ.

In summary, our study suggests that the new *PDGFRA* p.K455_N468delinsN splice mutation is potentially oncogenic. Additionally, we show that kinase inhibitors are potential treatment choices for GBM patients harboring this new mutation. However, as GBM is molecularly heterogeneous^53^, further combined clinical cancer genome analyses and in vitro genome editing studies are needed to completely characterize other VUS, thereby improving therapeutic interventions, particularly on molecularly targeted drugs.

## Methods

### Histological examination of GBM specimens

Resected specimens were fixed with phosphate-buffered neutral 10% formalin within 24 h and routinely processed for paraffin embedding (FFPE), sectioning, hematoxylin and eosin (H&E) staining, and immunohistochemistry (IHC) using antibodies against PDGFRα (C-9, Santa Cruz Biotechnology, Dallas, TX, USA) and the Envision FLEX High pH K8000 system (DAKO, Glostrup, Denmark). All tumors were classified according to the WHO classification 2016 by board-certified pathologists.

### Fluorescence in situ hybridization study of *PDGFRA*

*PDGFRA* fluorescent in situ hybridization (FISH) was performed on 4-μm-thick FFPE tissue sections of the three GBM cases. Bacterial artificial chromosome clone RP11-231C18 (*PDGFRA* gene, 4q12) was used as the FISH probe and CHR4-10-GR was used as the control probe (4p11) (both from Empire Genomics, Williamsville, NY, USA). The sections were counterstained with 4,6-diamidino-phenyl-indole and the images were captured with a fluorescence microscope (BX51, Olympus, Tokyo, Japan).

### DNA and mRNA sequencing of the clinical specimens

The clinical specimens were subjected to DNA and mRNA sequencing. Briefly, DNA and mRNA were extracted from FFPE tissues using the Maxwell 16 FFPE Tissue LEV DNA Purification Kit and Maxwell 16 LEV RNA FFPE Purification Kit (Promega, Madison, WI, USA), respectively. For DNA sequencing, comprehensive cancer panel analysis was performed using NGS as previously described^6^. For mRNA sequencing, the *PDGFRA* gene was amplified using RT-PCR as described below, and the PCR products were purified using the Exo-CIP Rapid PCR Cleanup Kit (New England Biolabs-NEB, Ipswich, MA, USA) and sequenced at GENEWIZ Japan Corp (Kawaguchi, Japan).

### Plasmid construction

For CRISPR/Cas9 experiments, the all-in-one CRISPR/Cas9 vector (pX330A-1 × 2, #58766, Addgene, Watertown, MA, USA) expressing the Cas9 nuclease and the relevant single-guided RNAs (sgRNAs) was used. The sequences of the sgRNAs are listed in Table S2. The sequences were annealed and cloned into the pX330A-1 × 2 vector as reported earlier^54^. The donor vector for *PDGFRA* gene editing was constructed based on the mNeonGreen cDNA, 5’ and 3’ homology arms (amplified using PCR from mNeonGreen-mTurquoise2; Addgene, #98886) and the genomic DNA from HEK293T cells using the primers listed in Table S3. The fragments were then inserted into a pCRIS-PITChv2-FBL backbone (Addgene, #63672) via DNA assembly (NEBuilder HiFi DNA Assembly Master Mix, NEB). To excise the donor cassette by micro-homology-mediated end joining, the pX-EGFP-g1 (Addgene, #107273) was used as a Cas9 expression vector.

To construct a PDGFRα expression vector, *PDGFRA* cDNA was amplified from the plasmid pDONR223-*PDGFRA* (Addgene, #23892) and inserted into a pCMV6-XL5 vector (OriGene Technologies, Rockville, MD, USA) between the HindIII and SalI restriction sites. As the *PDGFRA* cDNA in pDONR223-*PDGFRA* was mutated at position 260 (p.M260I, c.780G > A), the mutation was reconstituted to the wild-type form using a KOD-Plus-Mutagenesis Kit (TOYOBO, Osaka, Japan).

Additionally, the single-strand annealing (SSA) assay reporter plasmid was constructed using the plasmid pCAG-EGxxFP (Addgene, #50716); the wild-type genomic fragment containing the sgRNA target sites of *PDGFRA* was amplified and inserted into the pCAG-EGxxFP between the EcoRI and BamHI restriction sites.

### Cells and cell culture

HEK293T cells were obtained from the JCRB Cell Bank (Osaka, Japan) and maintained in Dulbecco’s Modified Eagle’s Medium (Thermo Fisher Scientific, Waltham, MA, USA) supplemented with 2 mM glutamine, 100 U/mL penicillin, 100 μg/mL streptomycin, and 10% fetal bovine serum at 37 °C with 95% air and 5% CO_2_.

### SSA assay

HEK293T cells (4 × 10^4^ cells/well) were seeded into a 96-well plate the day before transfection. The *PDGFRA*-targeted sgRNA and Cas9 expression (pX330A-1 × 2) and SSA reporter (pCAG-EGxxFP-*PDGFRA*) vectors were transfected using Lipofectamine 3000 (Thermo Fisher Scientific). The GFP-positive cells were observed under a fluorescence microscope (Axio Observer, Carl Zeiss, Oberkochen, Germany).

### T7 endonuclease I (T7E1) assay

HEK293T cells (4 × 10^4^ cells/well) were seeded into a 96-well plate the day before transfection. The pX330A-1 × 2 and pCAG-EGxxFP-*PDGFRA* vectors were transfected according to the method described above. After 48 h, genomic DNA was extracted using a Wizard SV Genomic DNA Purification System (Promega). The DNA fragment around the target site was amplified using the primers listed in Table S2. PCR products were denatured in NEBuffer 2 (NEB) at 95 °C for 5 min and annealed at a ramp rate of −2 °C/s (95–85 °C) or −0.1 °C/s (85–25 °C). Annealed PCR products were incubated with T7 Endonuclease I (T7EI; NEB) at 37 °C for 15 min and analyzed by electrophoresis using 2% agarose gel. The densitometry analysis was performed using CS Analyzer 3.0 (ATTO, Tokyo, Japan). Gene modification was calculated using the formula: % gene modification = 100 × [1 − (1 − fraction cleaved)^1/2^].

### Genome editing using CRISPR/Cas9

HEK293T cells (3 × 10^5^ cells/well) were seeded into a six-well plate the day before transfection. The *PDGFRA*-targeted sgRNA and Cas9 expression and donor vectors were transfected using Lipofectamine 3000. After 7 days, mNeonGreen-positive clones were isolated via single-cell sorting (SH-800S, SONY, Tokyo, Japan). To excise the inserted cassette, the ps1 targeted Cas9 expression vector (pX-EGFP-g1) was transfected into a selected clone (1D3) using Lipofectamine 3000. After 7 days, mNeonGreen-negative clones were isolated via single-cell sorting. Sorted cells were genotyped and sequenced as described below.

### Genomic PCR and DNA sequencing

Genomic DNA from cultured cells (5–10 × 10^5^ cells) was extracted using the Wizard SV Genomic DNA Purification System (Promega). Genomic PCR was performed on a Veriti 96-well Thermal Cycler (Thermo Fisher Scientific) using the primers listed in Table S3. The PCR products were detected by electrophoresis using 2% agarose gels. For DNA sequencing, the PCR products were purified using the Exo-CIP Rapid PCR Cleanup Kit (NEB) and sequenced at GENEWIZ Japan Corp.

### NGS analysis

The mutated *PDGFRA* was amplified from the genomic DNA of clones and sequenced on a MiSeq platform (Illumina, San Diego, CA, USA). Briefly, the DNA libraries were prepared from genomic PCR products using the GenNext NGS Library Prep Kit (TOYOBO), and TruSeq DNA Single indexes (Illumina). DNA libraries were purified using solid-phase paramagnetic beads (AMPure XP, Beckman Coulter, Brea, CA, USA).

### RT-PCR analysis of mRNA

Total RNA was extracted using the ReliaPrep RNA Cell Miniprep System (Promega) and converted into cDNA using the ReverTra Ace qPCR RT Kit & Master Mix (TOYOBO). mRNA expression was quantified via RT-PCR using the LightCycler 480 system (Roche Diagnostics, Basel, Switzerland), the THUNDERBIRD qPCR Mix (TOYOBO), the primers, and the Affinity Plus qPCR Probes (Integrated DNA Technologies, Coralville, IA, USA) listed in Tables S3 and S4. All reactions were performed in triplicate. *GAPDH* was used as the reference gene. The average of three threshold cycle values for the target and reference genes was calculated and gene expression was analyzed using the comparative Ct method.

### Cell cycle analysis

Cells were trypsinized and fixed in 70% ethanol at −20 °C. The fixed cells were washed with phosphate-buffered saline and incubated with FxCycle PI/RNase Staining Solution (Thermo Fisher Scientific) for 30 min at room temperature (22–25 °C) in the dark. The DNA content was analyzed using an Attune Nxt flow cytometer (Thermo Fisher Scientific).

### Drug sensitivity and proliferation assays

The *PDGFRA* mutant and isogenic control clones were seeded into 96-well plates (5000 cells/well) and allowed to adhere overnight. Cell Counting Kit-8 (WST-8, Dojindo, Kumamoto, Japan) was used to assess cell viability/proliferation. To assess drug sensitivity, culture media containing drugs at concentrations ranging from 0.5 to 8 μM was added to cells the day after seeding. Cell viability was evaluated using the WST-8 kit 4 d after treatment. The drugs used were as follows: temozolomide (Tokyo Chemical Industry, Tokyo, Japan), lenvatinib (Cayman Chemical, Ann Arbor, MI, USA), crenolanib (Abcam, Cambridge, UK), abemaciclib, and palbociclib (LKT Laboratories, St. Paul, MN, USA).

### Colony formation assay

Cloned cells were seeded into a 96-well plate (2000 cells/well), and the colony formation assay was performed after 4 d of culture using a Quantitative 3D Cell Culture Colony Assay Kit (Nippon Medical & Chemical Instruments, Osaka, Japan).

### Trypan blue exclusion assay

Cells were harvested and re-suspended in culture medium along with 0.4% trypan blue solution. Cell numbers and viability were examined using a cell counter (TC-20, Bio-Rad, Hercules, CA, USA).

### Sample preparation for immunoprecipitation

Wild-type or PDGFRα-mutated HEK293T cells were lysed using a Mem-PER Plus membrane protein extraction kit (Thermo Fisher Scientific). The lysates were fractionated using SDS-PAGE. Protein fractions were incubated with mouse anti-PDGFRα monoclonal antibodies (sc-398206, Santa Cruz Biotechnology), and the immunocomplex was captured with protein G beads (Tamagawa Seiki Co. Ltd., Nagano, Japan). The eluted samples were used for western blotting as described below.

### Western blotting

Cells were lysed using the Mem-PER Plus membrane protein extraction kit (Thermo Fisher Scientific). The lysates were fractionated using SDS-PAGE and transferred onto PVDF membranes. The membranes were blocked with 5% non-fat dried milk in TBS (pH 7.6) with 0.1% Tween 20 and incubated with the relevant primary antibodies diluted in Can Get Signal solution 1 (TOYOBO). Afterward, membranes were incubated with horseradish peroxidase-conjugated goat anti-rabbit or anti-mouse antibodies (Cell Signaling Technology, Danvers, MA, USA). Protein expression was detected using the SuperSignal West Pico chemiluminescent substrate (Thermo Fisher Scientific) or Clarity Max Western ECL Substrate (Bio-Rad). Densitometry analysis was performed using the CS Analyzer 3.0 (ATTO). The following antibodies were used in this assay (all from Cell Signaling Technology): anti-CDK4 (#12790), anti-CDK6 (#13331), anti-phospho-GSK3β (#9323), anti-GSK3β (#12456), anti-cyclin D1 (#55506), anti-c-Myc (#18583), anti-p-Akt (Thr 308; #4056), anti-p-Akt (Ser 473; #4058), anti-Akt (#4691), anti-p-Erk (Thr 202/Tyr 204; #4376), anti-Erk (#4695), anti-β-actin (#4970). PDGFRα expression was analyzed using mouse anti-PDGFRα monoclonal antibodies (sc-398206, Santa Cruz Biotechnology) and phosphorylated PDGFRα was detected using anti-phosphotyrosine antibodies (4G10, Merck Millipore, Burlington, MA, USA). The amounts of phosphorylated proteins were normalized to those of total proteins. For equal loading evaluation in the blotting, beta-actin expression was monitored.

### Overexpression of PDGFRα

HEK293T cells were seeded (7.5 × 10^6^ cells) into 90 mm dishes and transfected with wild-type or mutated PDGFRα expression vectors using Lipofectamine 3000. Forty-eight h after, the transfected cells were lysed using the Mem-PER Plus membrane protein extraction kit (Thermo Fisher Scientific), and proteins were visualized via fluorescent western blotting. Briefly, proteins were resolved by SDS-PAGE and transferred onto PVDF membranes. Next, membranes were blocked and incubated with antibodies using the protocol described above. The anti-PDGFRα (#5214, Cell Signaling Technology) and anti-phosphotyrosine (4G10) antibodies were used as the primary antibodies, and IRDye 680RD donkey anti-rabbit IgG and IRDye 800CW donkey anti-mouse IgG (LI-COR, Lincoln, NE, USA) were used as the secondary antibodies. Fluorescence detection and quantification were performed using Odyssey CLx (LI-COR).

### Statistical analysis

All experiments were performed 3–6 times independently. All data are presented as the mean ± SE. Statistical significance was determined using the unpaired one-tailed Student's *t*-test, and results with p < 0.05 were considered statistically significant.

### Ethical approval

The experiments based on clinical samples were approved by the Ethics Committee for Clinical and Epidemiologic Research, Kagoshima University. Written informed consent was obtained from each participant. Participants younger than 20 years of age were not included in this study. All research was performed in accordance with relevant guidelines/regulations, and research involving human research participants were performed in accordance with the Declaration of Helsinki.

## Supplementary Information

Supplementary Information.

## Data Availability

All data generated or analysed during this study are included in this published article (and its Supplementary Information files).
