# Supplementary material for: An oncogenic splice variant of PDGFRα in adult glioblastoma as a therapeutic target for selective CDK4/6 inhibitors
Source: Sci Rep. 2022 Jan 24;12:1275. doi: 10.1038/s41598-022-05391-9 (PMC8786844; doi:10.1038/s41598-022-05391-9)
Supplement: Supplementary file 1 — Supplementary Information. [file 41598_2022_5391_MOESM1_ESM.pdf]

Supplementary Figures and Tables

An Oncogenic Splice Variant of PDGFRα in Adult Glioblastoma as a Therapeutic Target for Selective CDK4/6 Inhibitors

Taiji Hamada,Toshiaki Akahane, Seiya Yokoyama, Nayuta Higa, Mari Kirishima, Kei Matsuo, Michiko Shimokawa, Koji Yoshimoto, Akihide Tanimoto

Supplementary Table S1

GBM cases with *PDGFRA* gene amplification and/or mutations

No	Location	Age	Sex	TC (%)	VAF (%)	Mutation	CN	COSMIC ID	JAX	OncoKB
1	Rt temporal lobe	70	M	50	69	p.Gly286Arg	40	6354900	NR	NR
2	Lt caudate nucleus	67	M	60	87	p.Asn468Ser	84	2155032	NR	NR
3	Rt parietal lobe	77	F	70	39	p.D842_I843delinsV	4	405285	GOF (p.D842_I843delinsIM)	GOF (p.D842_I843delinsIM)
4	Rt radiate crown	76	M	60	84	p.Asn468Ser	19	2155032	NR	NR
5	Lt radiate crown	75	M	70	69	p.Ala396Pro	32	-	NR	NR
6	Lt temporal lobe	62	M	70	28	p.Cys235Ser	50	3604496	VUS	NR
				70	40	p.Pro345Ser		7697761	Predicted GOF	NR
7	Rt temporal lobe	81	F	80	92	p.Trp447Cys	73	3409361	NR	NR
8	Lt cerebellum	67	M	50	17	p.Leu275Ser	30	-	NR	NR
9	Left frontal lobe	86	M	60	76	p.Asn468Ser	26	2155032	NR	NR
10	Rt frontal lobe	76	F	50	43	p.Ile363Phe	3	-	NR	NR
				50	49	p.Asp842His		734174	GOF (p.Asp842Val)	GOF (p.Asp842Val)
11	Rt frontal lobe	72	M	40	60	p.Pro343Ser	63	6934874	NR	NR
				40	60	p.Leu354Val		-	NR	NR

M, male; F, female; TC, tumor content; VAF, variance unknown significance; CN, copy number; NR, no registration; GOF, gain of function

Supplementary Figure S1

	position		target sequence		sequence information				number of target sites		
	start - end	+ −	20mer+PAM (total 23mer)		GC% of 20mer	Tm of 20mer	TTTT in 20mer	restriction sites	20mer +PAM	12mer +PAM	8mer +PAM
	304 - 326	+	CACTCATTGCCATGACTCTCAGG	[gRNA]	50.00 %	70.31 °C	-		1 [detail]	20 [detail]	5032 [detail]
	310 - 332	+	TTGCCATGACTCTCAGGAATTGG	[gRNA]	45.00 %	70.73 °C	-		1 [detail]	34 [detail]	7187 [detail]
	313 - 335	-	CCATGACTCTCAGGAATTGGCCC	[gRNA]	55.00 %	75.11 °C	-		1 [detail]	55 [detail]	6828 [detail]
	323 - 345	+	CAGGAATTGGCCCTATACTTAGG	[gRNA]	45.00 %	71.53 °C	-		1 [detail]	5 [detail]	3042 [detail]
	333 - 355	-	CCCTATACTTAGGCCCTTTTTCT	[gRNA]	35.00 %	67.32 °C	-		1 [detail]	6 [detail]	3746 [detail]
	334 - 356	-	CCTATACTTAGGCCCTTTTTCTC	[gRNA]	40.00 %	68.55 °C	-		1 [detail]	4 [detail]	2496 [detail]
	346 - 368	-	CCCTTTTTCTCTCTAGATGTAAT	[gRNA]	25.00 %	58.98 °C	-	XbaI	1 [detail]	146 [detail]	20632 [detail]
	347 - 369	-	CCTTTTTCTCTCTAGATGTAATA	[gRNA]	25.00 %	59.06 °C	-	XbaI	1 [detail]	77 [detail]	34350 [detail]
	361 - 383	+	GATGTAATAATGAAACTTCCTGG	[gRNA]	30.00 %	59.76 °C	-		1 [detail]	30 [detail]	6953 [detail]
**sgRNA6**	371 - 393	+	TGAAACTTCCTGGACTATTTTGG	[gRNA]	35.00 %	65.50 °C	-		1 [detail]	25 [detail]	4161 [detail]
**sgRNA4**	379 - 401	-	CCTGGACTATTTTGGCCAACAAT	[gRNA]	40.00 %	68.21 °C	-	EaeI MscI	1 [detail]	26 [detail]	3167 [detail]
**sgRNA1**	394 - 416	-	CCAACAATGTCTCAAACATCATC	[gRNA]	35.00 %	63.13 °C	-		1 [detail]	11 [detail]	3902 [detail]
**sgRNA5**	398 - 420	+	CAATGTCTCAAACATCATCACGG	[gRNA]	35.00 %	62.62 °C	-		1 [detail]	43 [detail]	6295 [detail]
**sgRNA2**	418 - 440	+	CGGAGATCCACTCCCGAGACAGG	[gRNA]	65.00 %	77.32 °C	-		1 [detail]	5 [detail]	4633 [detail]
	425 - 447	-	CCACTCCCGAGACAGGAGTACCG	[gRNA]	65.00 %	78.05 °C	-		1 [detail]	12 [detail]	2148 [detail]
**sgRNA3**	428 - 450	+	CTCCCGAGACAGGAGTACCGTGG	[gRNA]	65.00 %	78.05 °C	-	BtgI	1 [detail]	2 [detail]	635 [detail]
	430 - 452	-	CCCGAGACAGGAGTACCGTGGAG	[gRNA]	60.00 %	75.64 °C	-	BtgI	1 [detail]	26 [detail]	9471 [detail]
	431 - 453	+	CCGAGACAGGAGTACCGTGGAGG	[gRNA]	65.00 %	78.15 °C	-	BtgI	1 [detail]	4 [detail]	692 [detail]
	431 - 453	-	CCGAGACAGGAGTACCGTGGAGG	[gRNA]	60.00 %	77.54 °C	-	BtgI	1 [detail]	28 [detail]	9968 [detail]
	432 - 454	+	CGAGACAGGAGTACCGTGGAGGG	[gRNA]	60.00 %	76.25 °C	-	BtgI	1 [detail]	4 [detail]	1412 [detail]
	445 - 467	-	CCGTGGAGGGCCGTGTGACTTTC	[gRNA]	60.00 %	77.33 °C	-	BtgI	1 [detail]	15 [detail]	7255 [detail]

Target mutation (c.1403A>G)

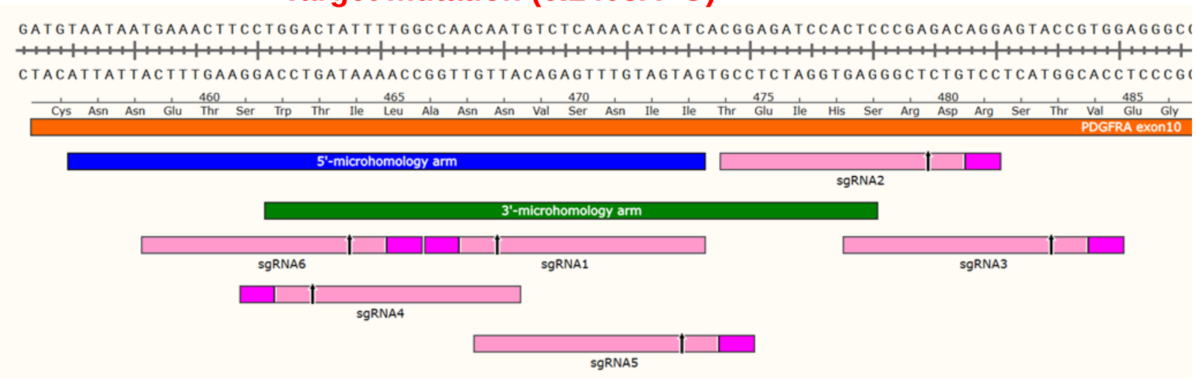

Figure S1. Selection of sgRNA for the insertion of the targeting and excision markers into *PDGFRA* gene.

Single-guided RNAs were designed using CRISPRdirect (https://crispr.dbcls.jp/); six sgRNA candidates for the introduction of the DSB near the target mutation of *PDGFRA* were selected.

Supplementary Figure S2

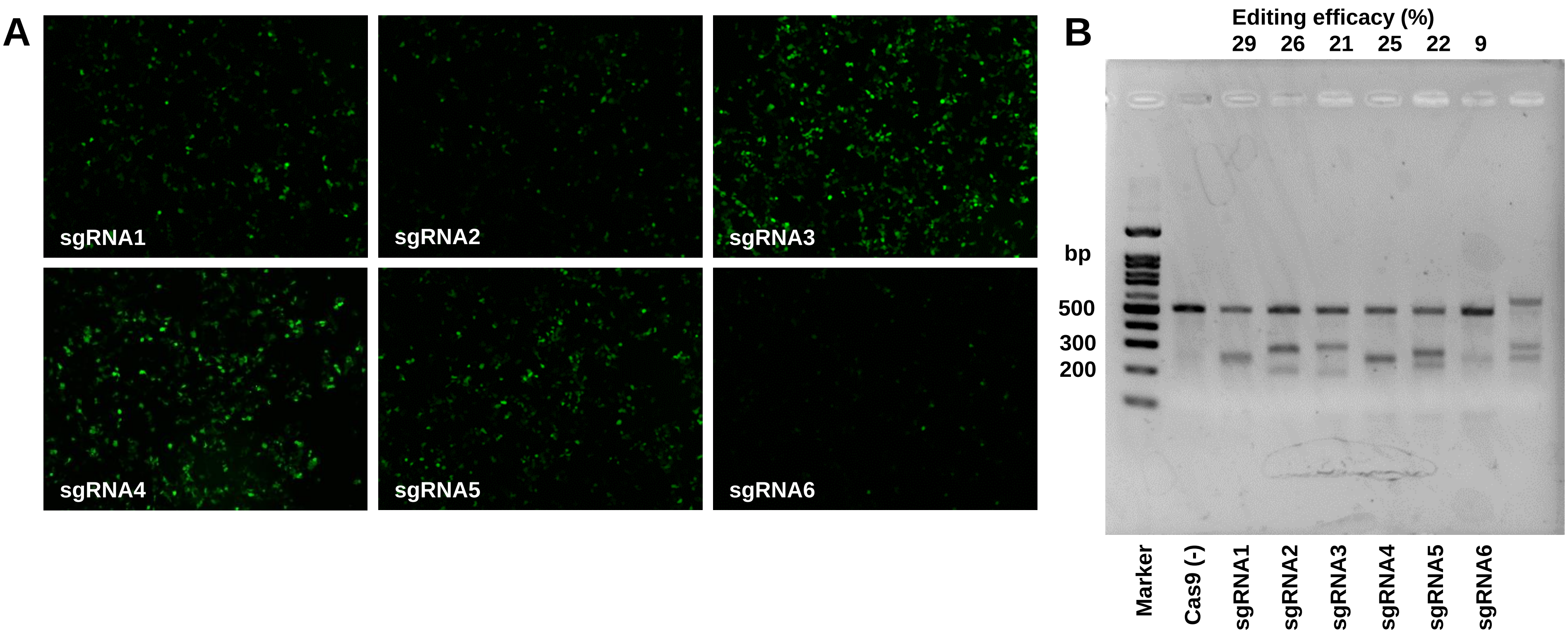

Figure S2. Validation of the PDGFRA sgRNAs.

A) The SSA assay showed that sgRNA3 and sgRNA4 exhibited the higher DSB activity, as per the monitoring of GFP signals. **B)** The T7E1 assay showed that almost comparable breaking activities were detected among the 6 sgRNAs.

Supplementary Figure S3

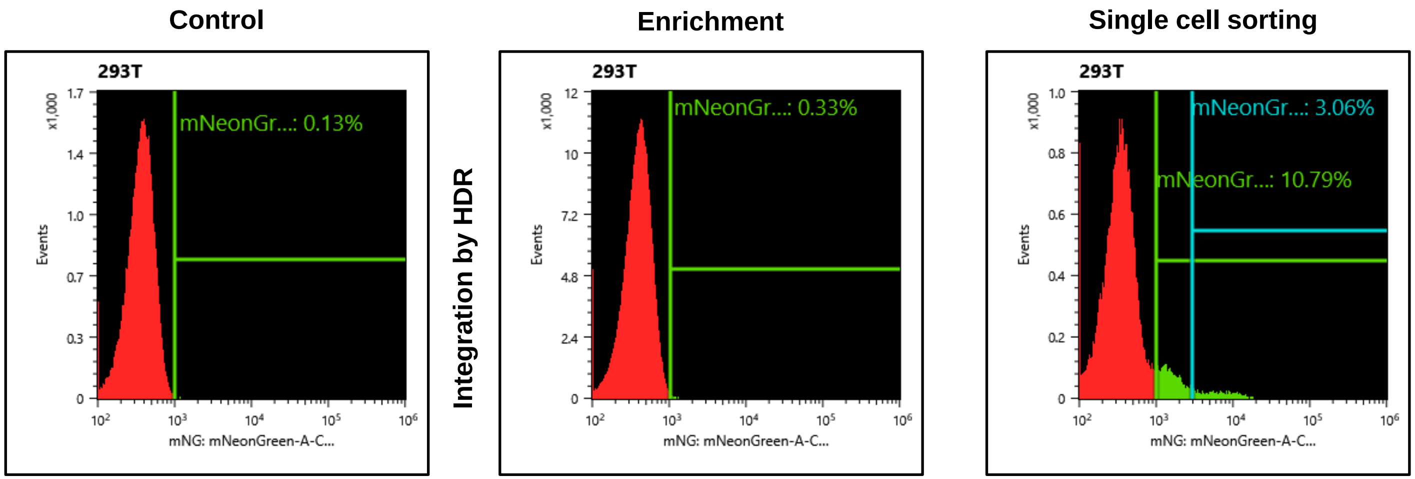

Figure S3. Sorting of mNeonGreen-positive cells: 1st editing.

After transfection with the Cas9 expression plasmid and donor vectors, mNeonGreen positive cells (Green) were enriched and isolated as single cells using a Cell sorter: among others, the 1D3 clone was obtained.

Supplementary Figure S4

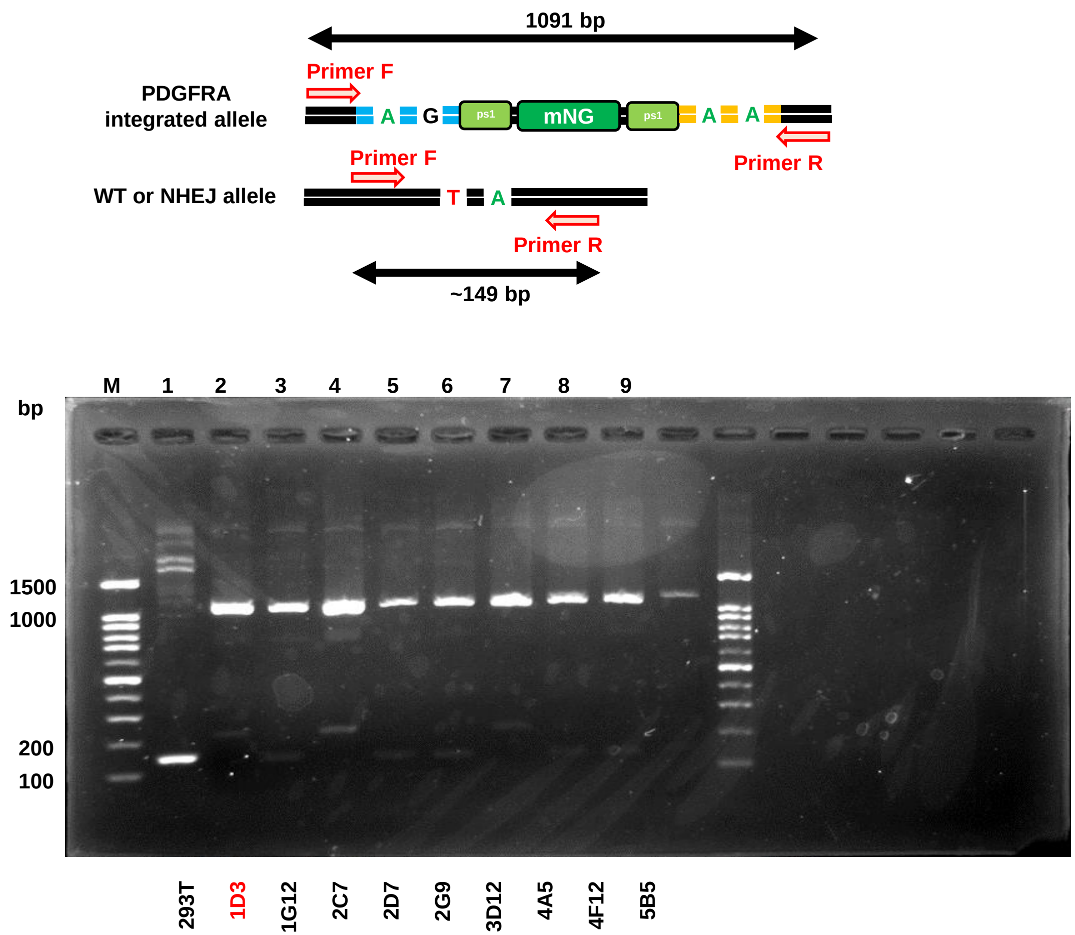

Figure S4. Schematic presentation of the insertion of the selection cassette and PCR-based genotyping.

A representative result of PCR-based genotyping for the identification of the insertion of the selection cassette in single clones. PCR products were analyzed using a 2% agarose gel: lane 1, parental HEK293T cells; lane 2, clone 1D3; and lanes 3 to 9, other clones.

Supplementary Figure S5

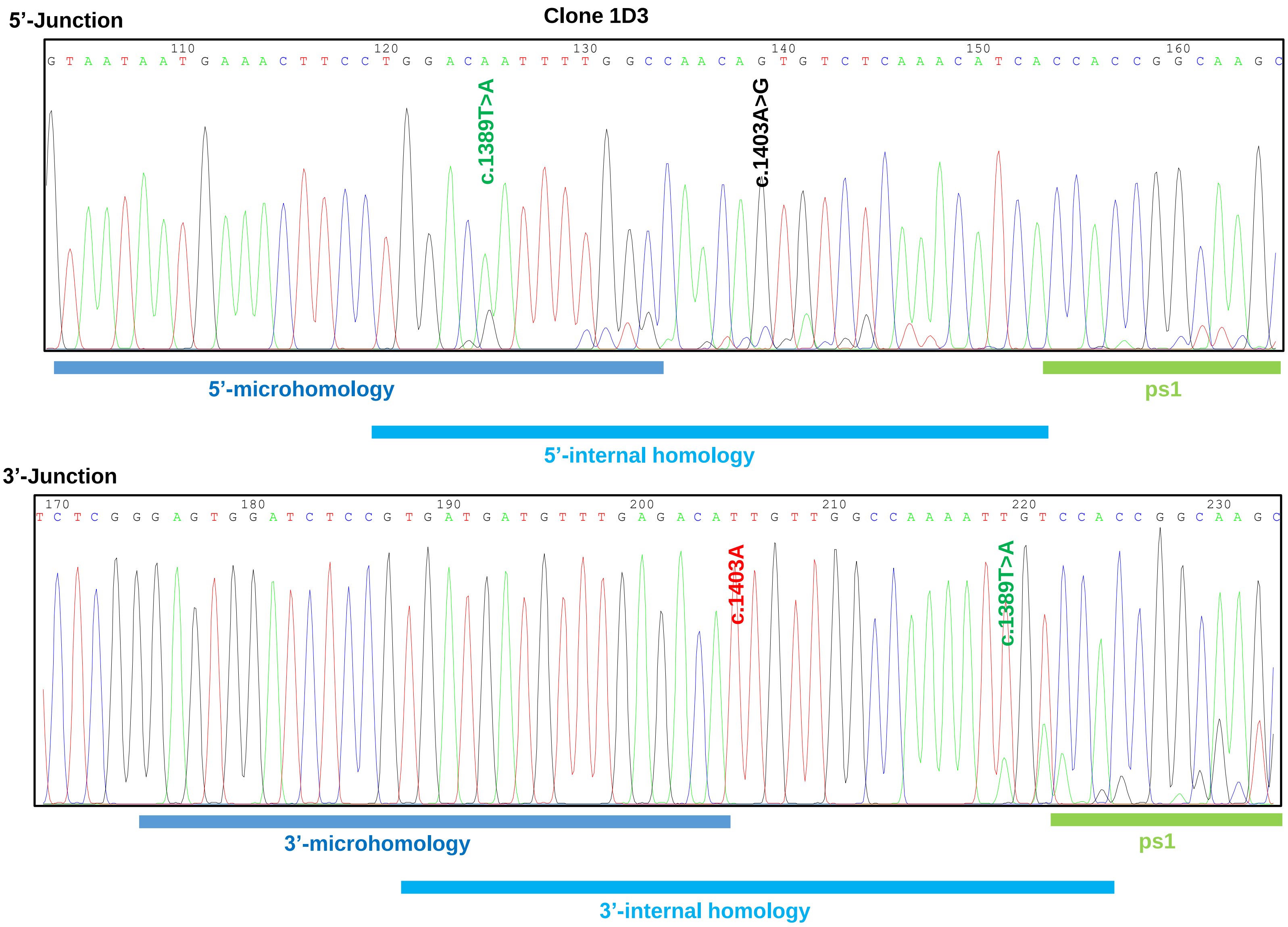

Figure S5. Sanger sequencing data of the clone 1D3.

Sanger sequencing revealed the correct insertion of the selection cassette in the clone 1D3.

Supplementary Figure S6

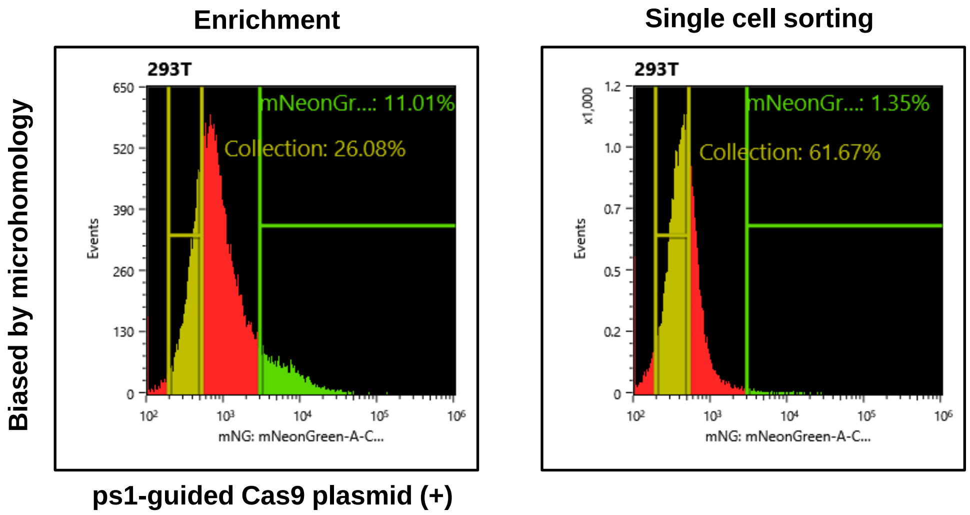

Figure S6. Sorting of mNeonGreen-negative cells: 2nd editing round.

The selection cassette was deleted out of the *PDGFRA* gene in clone 1D3. Using a cell sorter, mNeonGreen-negative cells were selected and cells harboring the target gene mutation as well as isogenic control cells were obtained.

Supplementary Figure S7

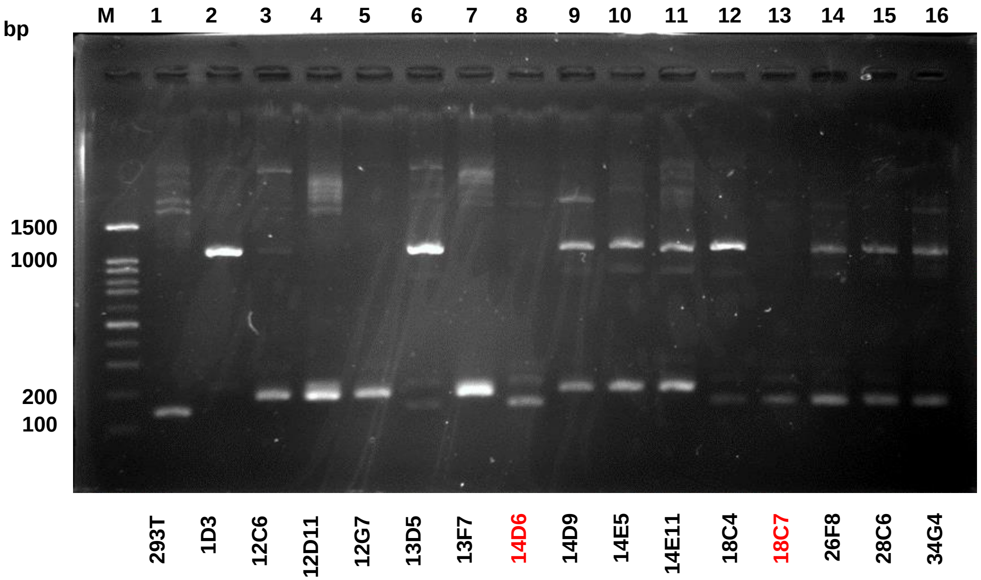

Figure S7. PCR-based analysis for the removal of the cassette in the selected clones.

Gel analysis of the edited clones. Lane 1, HEK293T parental cells; lane 2, clone 1D3; lane 8, clone 14D6 (precise mutation); lane 13, clone 18C7 (isogenic control). PCR bands from the selected clones revealed the removal of the cassette.

Supplementary Figure S8

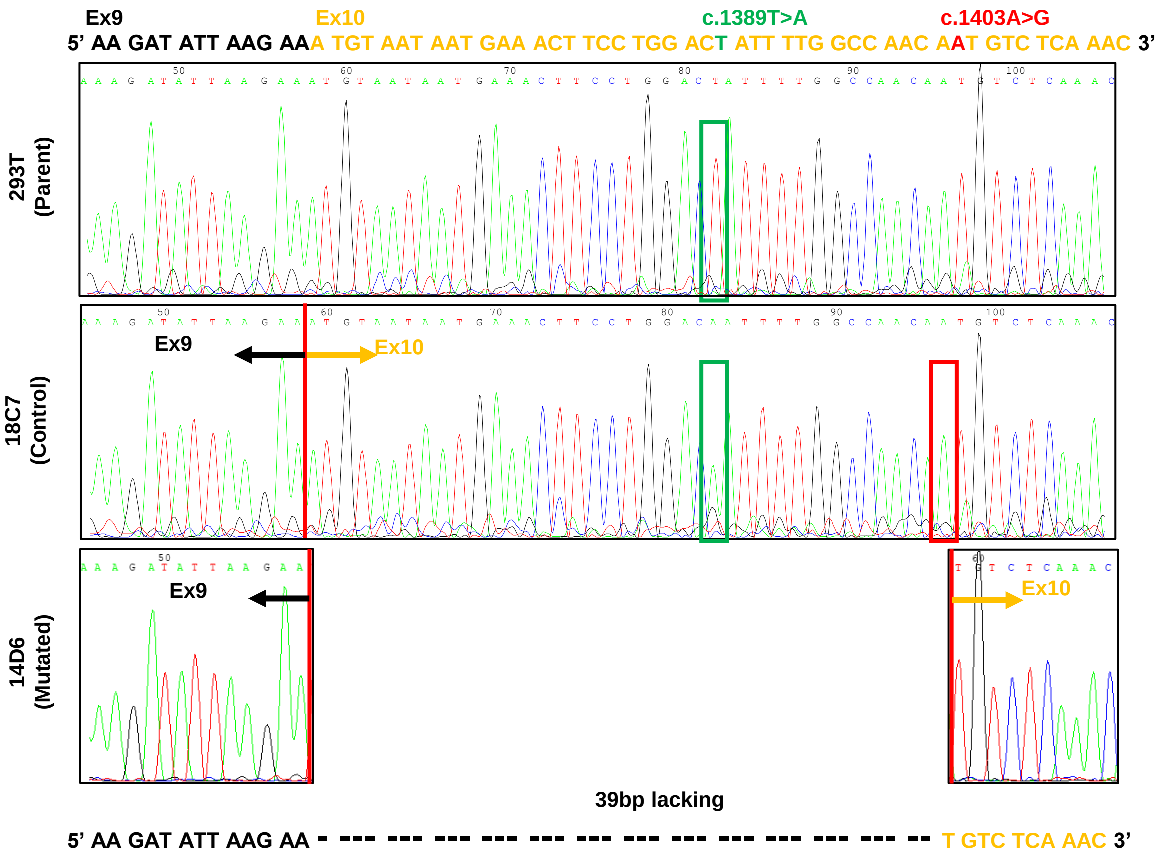

Figure S8. Structure of *PDGFRA* mRNA after the introduction of c.1403A>G mutation.

Sanger sequencing results of the established clones. Green and red boxes indicate the silent and targeted mutations, respectively. In clone 14D6, but not in control 18C7 (isogenic control) and parental cells, the first 39 bp of exon 10 were deleted due to the generation of a new splicing site at position1403 because of c.1403A>G mutation.

Supplementary Figure S9

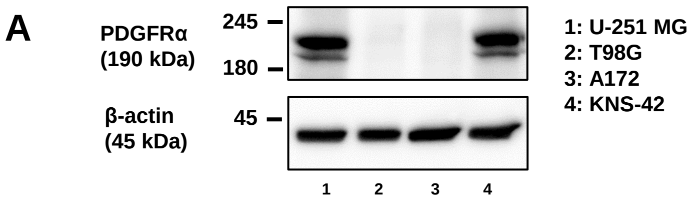

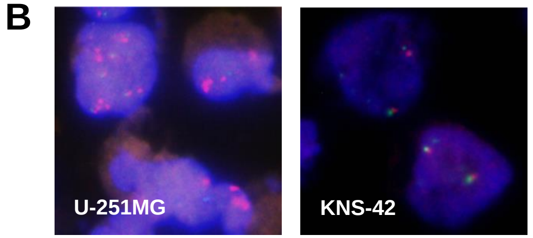

Cell lines	Signal ratio (PDGFRA /CEP4)	PDGFRA copy number (red)	CEP4 copy number (green)
U-251MG	2.07	6.0	2.9
KNS-42	1.0	2.23	2.23

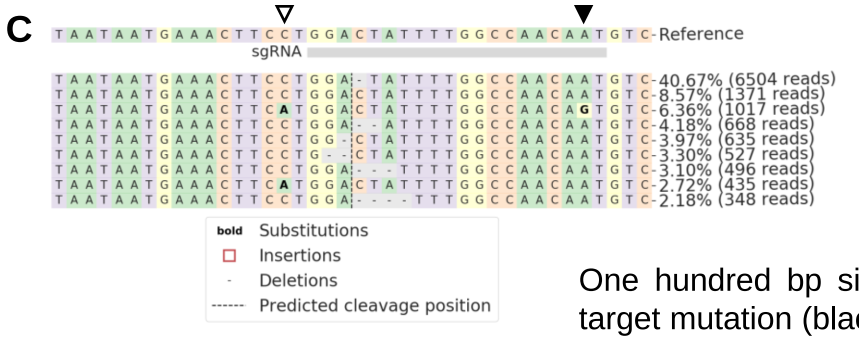

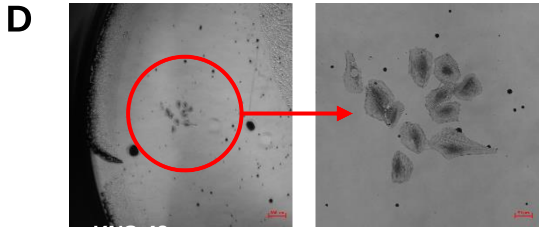

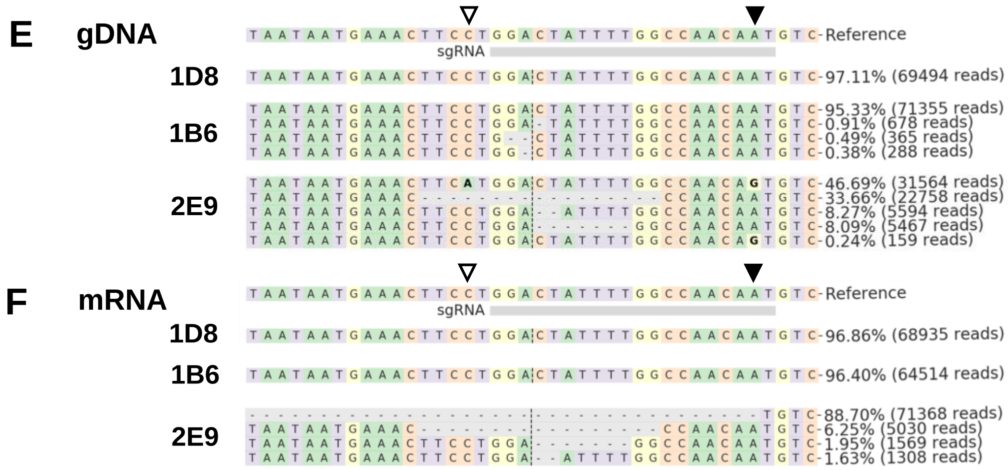

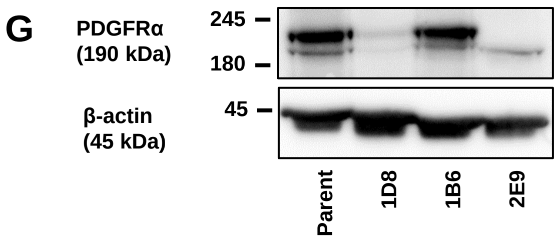

Figure S9. Glioblastoma cell lines with *PDGFRA* mutation
To select glioblastoma cell lines suitable for genome editing, western blotting of PDGFRα (A) and FISH (B) of *PDGFRA* were performed. KNS-42 cells, expressing high level of PDGFRα and having close to normal copy number, were selected.
One hundred bp single-stranded ODN with the silent mutation (white arrowhead) and the target mutation (black arrowhead), and Cas9 RNP were electroporated into KNS-42 cells, and the target mutation (6.36%) in the genomic DNA was confirmed by NGS (C). Edited KNS-42 cells were sorted after electroporation; however, the cloned cells did not expand after 3-week-subculture (D). U-251 MG cell line was edited by the same method, and the cloned cells with the mutation (2E9, 46.69%) and wildtype cloned cells (1D8, 97.11%; 1B6, 95.33%) were expanded (E). The 2E9 cells harbored the splice variant (p.K455_N468delinsN) (88.70%) (F). However, PDGFRα (G) were not sufficiently expressed in the parental wildtype 1D8 and mutated 2E9 clones. For uncropped blot sources of Figs. S9A and S9G, see Supplemental Fig. S14.

Supplementary Figure S10

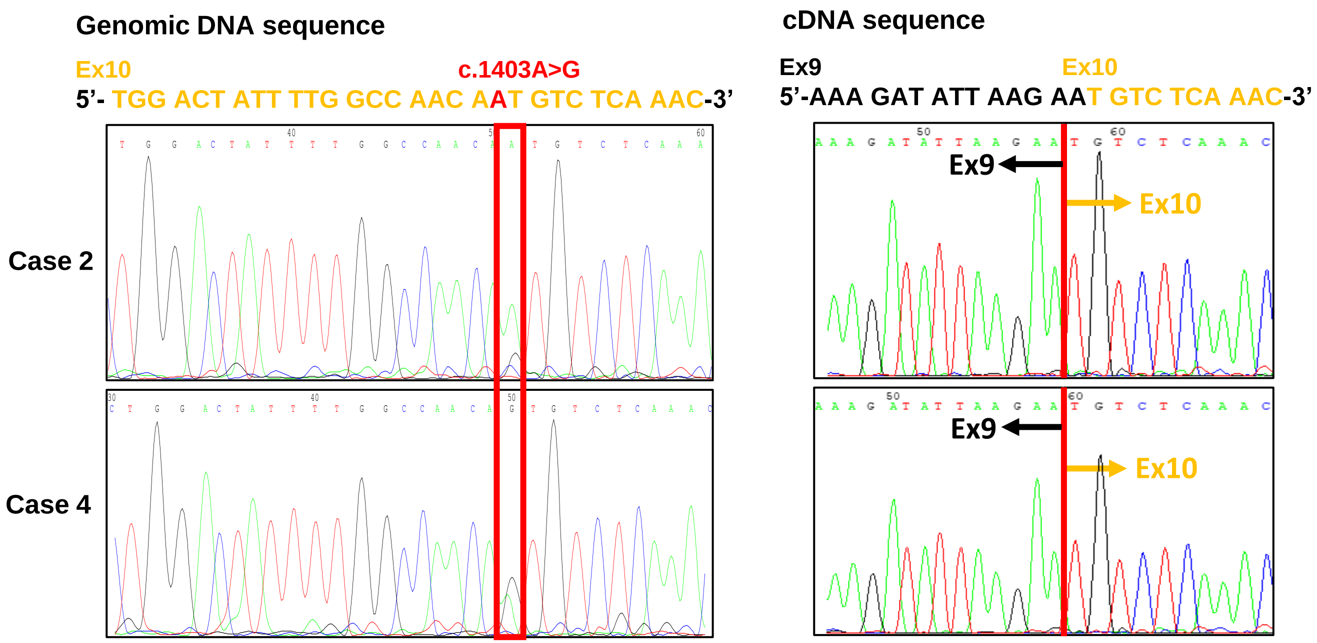

Figure S10. Genomic and mRNA sequencing of GBM clinical cases.

Genomic and mRNA sequences of *PDGFRA* were analyzed in the GBM cases. The c.1403A>G mutation and the subsequent *PDGFRA* splicing variant (and not the missense mutation p.N468S) was confirmed. Sanger sequencing (left) showed a main [A] peak at c.1403, but also a minor [G] peak in Case 1. Two peaks – main [G] and minor [A] peaks - were also detected in Case 2. As per the mRNA sequencing (right), only mRNA with the in-frame internal deletion was observed in both cases.

Supplementary Figure S11

Extended Figure 2C

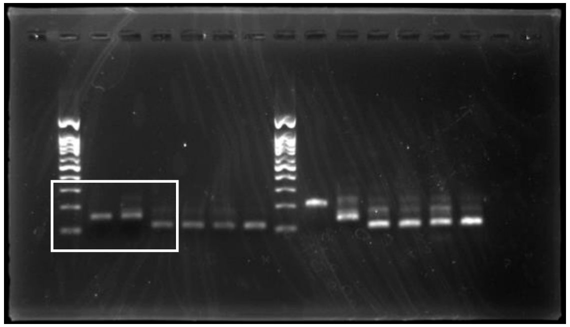

Extended Figure 4A

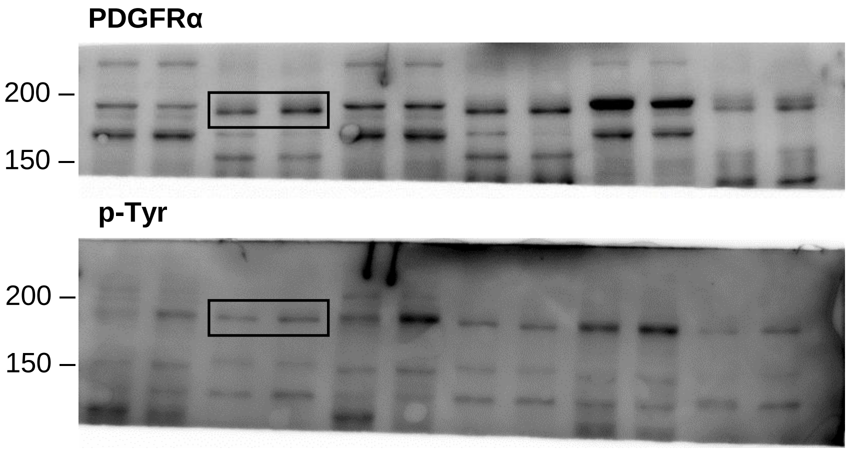

Extended Figure 4C

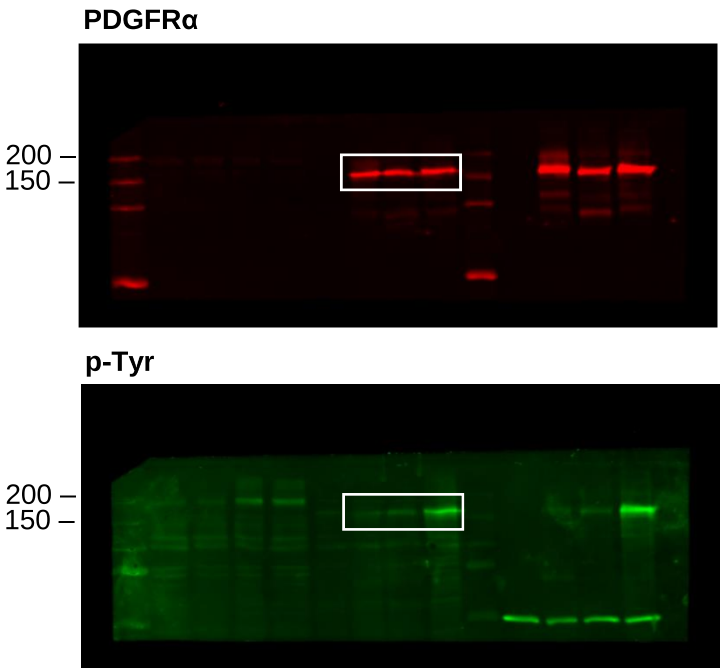

Figure S11. Extended data of Figs. 2C, 4A, and 4C.

Uncropped original gel electrophoresis and blot data of Figs. 2C, 4A, and 4C. The main-text figures are shown above each gel with a black outline to show the excerpted portions.

Supplementary Figure S12

Extened Figure 4D

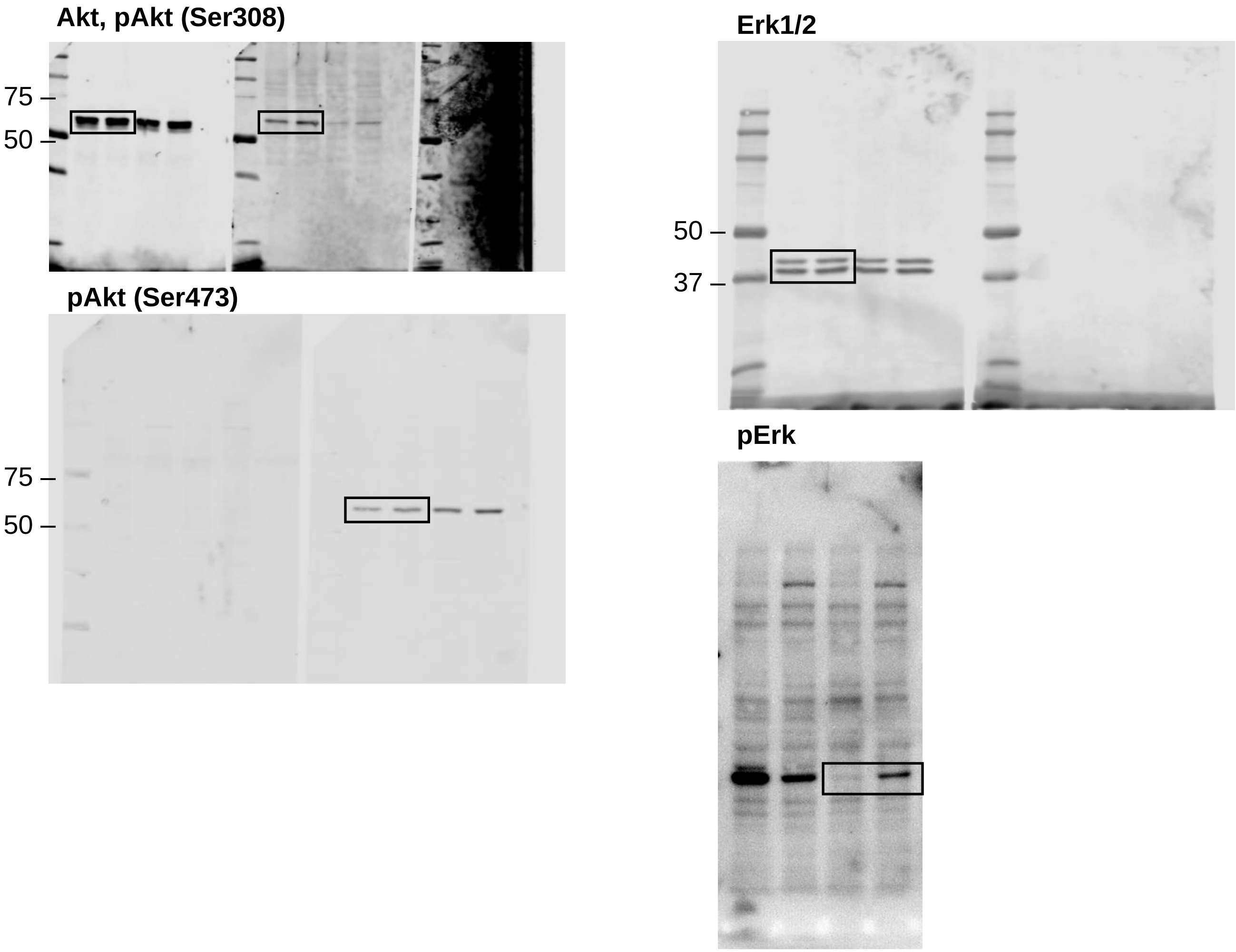

Figure S12. Extended data of Fig. 4D.

Uncropped original blot data of Fig. 4D. The main-text figures are shown above each gel with a black outline to show the excerpted portions.

Supplementary Figure S13

Extended Figure 5A

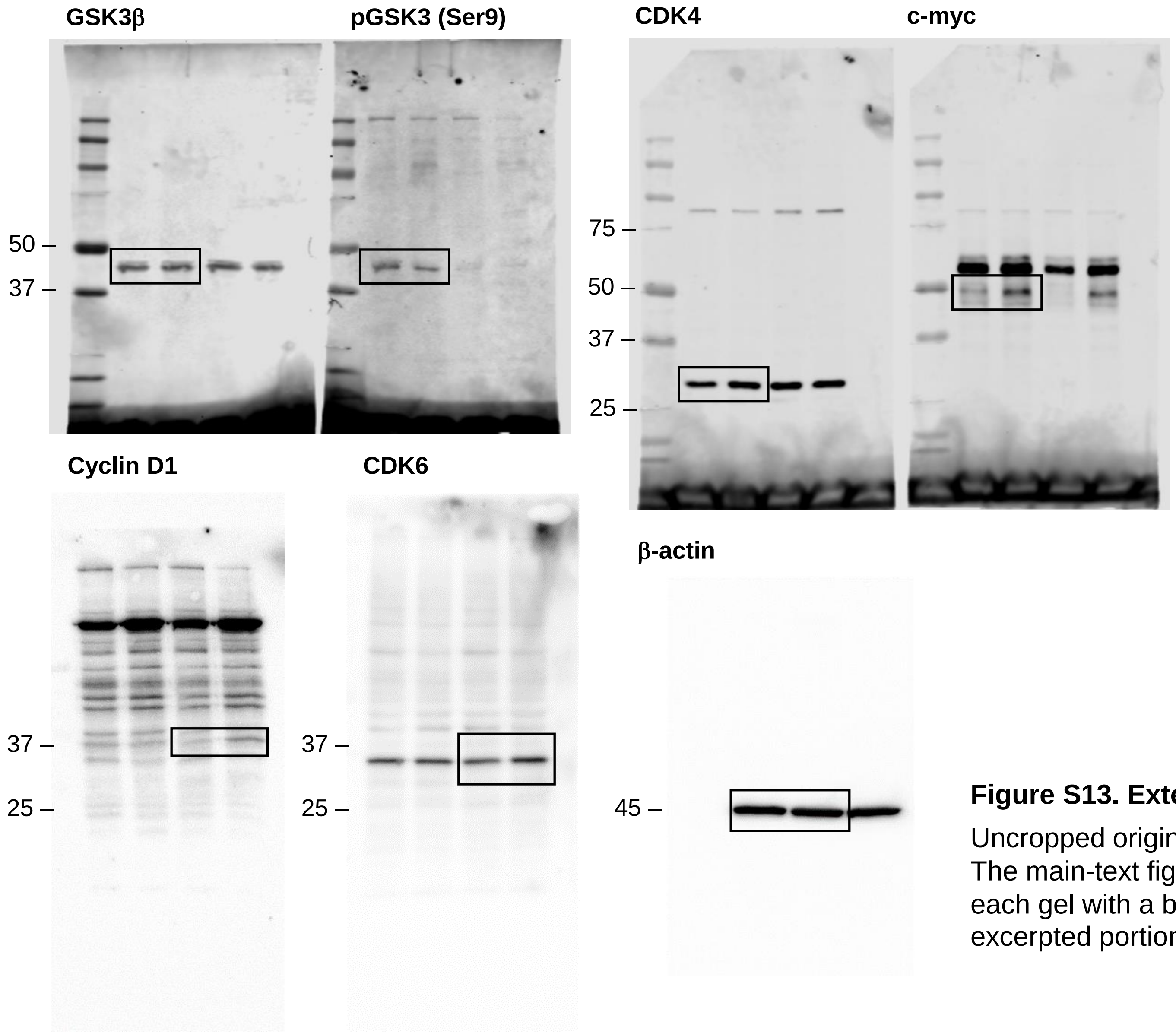

Figure S13. Extended data of Fig. 5A.

Uncropped original blot data of Fig. 5A. The main-text figures are shown above each gel with a black outline to show the excerpted portions.

Supplementary Figure S14

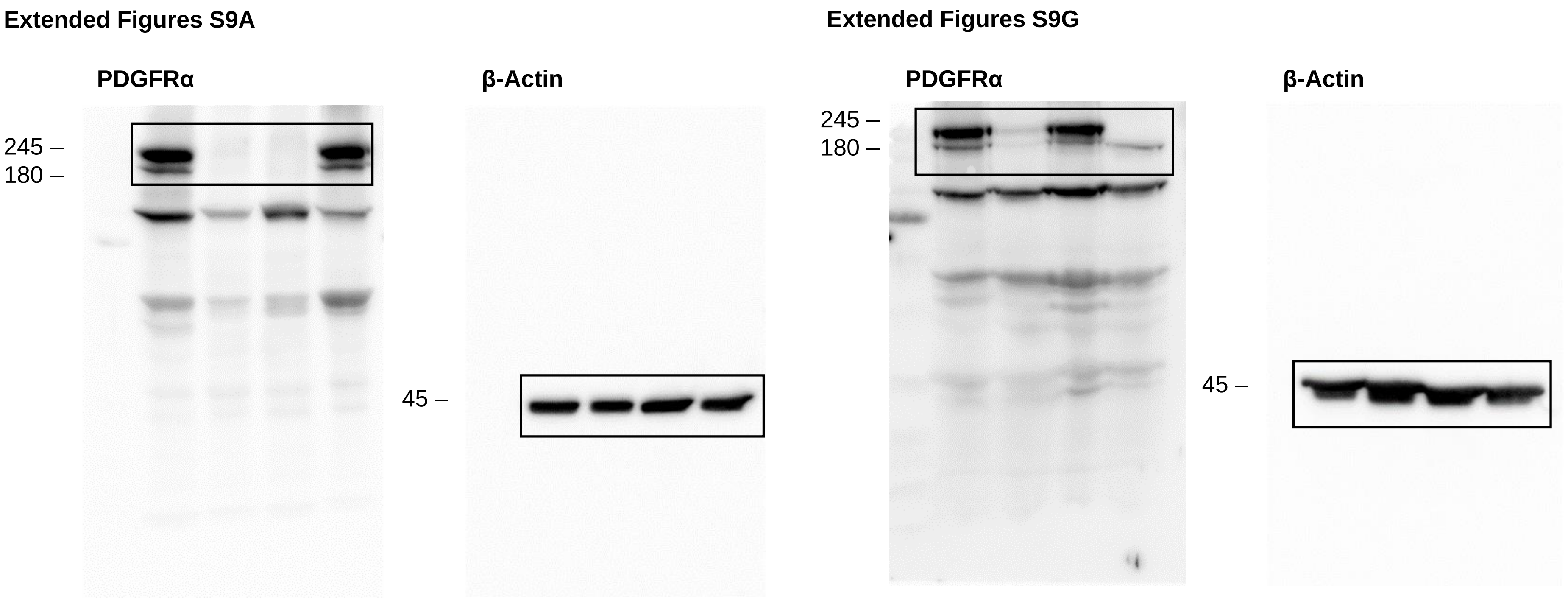

Figure S14. Extended data of Fig. S9A and S9G.

Uncropped original blot data of Figs. S9A and S9G. The main-text figures are shown above each gel with a black outline to show the excerpted portions.

Supplementary Table S2

Sequences of DNA oligonucleotides

	**Oligonucleotides**	**Sequences (5' – 3')**
***PDGFRA* gene editing**	sgRNA1_top	CACCGATGATGTTTGAGACATTGT
	sgRNA1_bottom	AAACACAATGTCTCAAACATCATC
	sgRNA2_top	CACCGCGGAGATCCACTCCCGAGAC
	sgRNA2_bottom	AAACGTCTCGGGAGTGGATCTCCGC
	sgRNA3_top	CACCGCTCCCGAGACAGGAGTACCG
	sgRNA3_bottom	AAACCGGTACTCCTGTCTCGGGAGC
	sgRNA4_top	CACCGATTGTTGGCCAAAATAGTCC
	sgRNA4_bottom	AAACGGACTATTTTGGCCAACAATC
	sgRNA5_top	CACCGCAATGTCTCAAACATCATCA
	sgRNA5_bottom	AAACTGATGATGTTTGAGACATTGC
	sgRNA6_top	CACCGTGAAACTTCCTGGACTATTT
	sgRNA6_bottom	AAACAAATAGTCCAGGAAGTTTCAC

Supplementary Table S3

Sequencing primers

	Primers	Sequences (5' – 3')
**Vector construction**		
**Donor**	Left homology arm_F	CCGCGTTACATAGCAAAGAAGGTCCAGGGCTCTCA
	Left homology arm_R	TGTTGGCCAAAATtGTCCAGGAAGTTTCATTATTA
	Right homology arm_F	TGGACaATTTTGGCCAACAaTGTCTCAAACATCAT
	Right homology arm_R	CAGCATTCTAGAGCAAGGGCTGCCTGGTTCATTTT
	mNeonGreen_F	CTGGACaATTTTGGCCAACAgTGTCTCAAACATCACCACCGGCAAGCTGCCCGTGCCCGCCACAAACTTCTCTC TGCTAAAGCAAGCAGGTGATGTTGAAGAAAACCCCGGGCCTGTCGCCACCATGGTGAGCAA
	mNeonGreen_R	tTGTTGGCCAAAATtGTCCACCGGCAAGCTGCCCGTGCCCGTCGACTTCTAGATTTAATTAACTCGAGCCTAGGGC CGGGATTCTCCTCCA
	pCRIS_F	TGCTCTAGAATGCTGATGGGCTAGCAAAATC
	pCRIS_R	TGCTATGTAACGCGGAACTCCATATATGGG
**PDGFRα overexpression**	*PDGFRA*cDNA_F	CAAAAGCTTGTACAAAAAAGTTGGCACCATG
	*PDGFRA*cDNA_R	AAAGTCGACCTACAGGAAGCTGTCTTCCACCAG
	*PDGFRA*mt_M260I_F	TgCTGGAAGAAATCAAAGTCCCAT
	*PDGFRA*mt_M260I_R	TTGTGATGCCTTTGCCTTTCACTT
	*PDGFRA*mt_E10del2_F	AgTGTCTCAAACATCATCACGGAGATC
	*PDGFRA*mt_E10del2_R	GTTGGCCAAAATAGTCCAGGAAGTTTC
	*PDGFRA*mt_D842V_F	GtCATCATGCATGATTCGAACTATGTGTCG
	*PDGFRA*mt_D842V_R	TCTGGCCAGGCCAAAGTCACAGATC
**SSA assay**	SSA_*PDGFRA*_F	TCAGCGAATTCTTTTTGAAGGGGTGGGAGATTGA
	SSA_*PDGFRA*_R	TAAGGATCCGGATTCAAAGTTCCGCCTGGG
**T7E1 assay**		
	PDGFRA-KI_F	GCTATGTAAACACTTAGCTTTCAGTTGTTCATGTG
	PDGFRA-KI_R	TTCTATATAACCCCTATCTGGGATTCAAAGTTCC

Supplementary Table S3 (continued)

Sequencing primers

	Primers	Sequences (5' – 3')
**Genomic PCR for cloning**		
	PDGFRA-gDNA_F	AGGAATTGGCCCTATACTTAGG
	PDGFRA-gDNA/mRNA_R	TTGGCGAAAGTCACACG
	PDGFRA-KI_F2	AATCAGTGTGTATTGCCCCG
	PDGFRA-KI_R2	TCTGGGATTCAAAGTTCCGC
**Messenger RNA analysis**		
	PDGFRA-mRNA_F	CCTGATATTGAGTGGATGATATGC
	PDGFRA-gDNA/mRNA_R	TTGGCGAAAGTCACACG

Supplementary Table S4

Sequences of hydrolysis probes

**Probes**	**Sequences (5’ – 3’)**
**Assay ID: Hs.PT.58.45699973**<br>**(Integrated DNA Technologies, Inc.)**	*PDFGRA* exons 22-23 (mixture of probe and primer pair)
***PDGFRA*-WT(A)_FAM**	TGAGACATTGTTGG
***PDGFRA*-MT(E10del2)_FAM**	TGAGACATTCTTAATATCT
